# Mid-infrared intracavity quartz-enhanced photoacoustic spectroscopy with pptv – Level sensitivity using a T-shaped custom tuning fork

**DOI:** 10.1016/j.pacs.2022.100330

**Published:** 2022-01-11

**Authors:** Jakob Hayden, Marilena Giglio, Angelo Sampaolo, Vincenzo Spagnolo, Bernhard Lendl

**Affiliations:** a Institute of Chemical Technologies and Analytics, Technische Universität Wien, Getreidemarkt 9, 1060 Vienna, Austria; b PolySense Lab - Dipartimento Interateneo di Fisica, University and Politecnico of Bari, Via Amendola 173, Bari, Italy

**Keywords:** QEPAS, Cavity enhanced spectroscopy, Trace gas sensing, Costum quartz tuning fork, Quantum cascade laser

## Abstract

Resonant optical power buildup inside a high finesse cavity is exploited to boost the sensitivity in quartz-enhanced photoacoustic spectroscopy (QEPAS) for CO, N_2_O and H_2_O detection, operating at a wavelength of 4.59 µm. A quartz tuning fork with T-shaped prongs optimized for QEPAS has been employed. Exploiting the high signal-to-noise ratio attainable with this tuning fork together with an optical power amplification of ~100 enabled by efficient optical feedback locking, limits of detection (3σ, 10 s integration) of 260 ppt and 750 ppt for CO and N_2_O have been reached.

## 1 Introduction

Since its first demonstration in 2002 [1], quartz-enhanced photoacoustic spectroscopy (QEPAS) has evolved into a widely employed technique for trace gas sensing. The popularity of QEPAS can be attributed to the very compact footprint of QEPAS sensors and small gas cell volumes [2], [3], the strong resonant enhancement of signals due to the high quality electro-mechanic resonance of the quartz tuning fork (QTF) transducer, and its acoustic quadrupole acoustic configuration yielding low sensitivity to ambient noise [4]. The concentration of gases detected with the QEPAS technique typically spans the range from percent to parts per billion (ppb). In particular, using quantum cascade lasers or other lasers emitting in the mid-infrared, strong fundamental ro-vibrational transitions of molecules can be targeted and limits of detection (LOD) between 1 part per million (ppm) and ~ 10 ppb can be achieved with a typically available optical power between 1 mW and 100 mW [4]. In the past years, significant improvements have been achieved for QEPAS through, amongst others, the development of custom designed QTFs with geometries optimized for photoacoustic sensing [5]. Compared to the standard 32.8 kHz-QTF, the prong spacing was increased, the resonance frequency was decreased and the electro-mechanical properties were optimized via the shape of the prongs of the QTF [6].

A common strategy to further increase the sensitivity of photoacoustic spectroscopy (PAS) and QEPAS is to increase the available optical power using high power lasers [7], [8], [9]. The optical power can be increased even further inside high finesse optical cavities, as was demonstrated for PAS [10], [11], [12], cantilever enhanced PAS where, for example, a LOD (3 σ, 10 s integration) of 225 part per trillion (ppt) was reported for acetylene [13] and QEPAS where a LOD of 300 ppt (1 σ, 4 s integration) for CO_2_ has been demonstrated [14], [15]. Recently an intracavity QEPAS (I-QEPAS) setup for CO detection at 4.59 µm has been reported [16]. The setup exploited a Fabry-Perot Brewster window cavity and a QEPAS spectrophone, composed of a 1.5 mm-prong spacing QTF with a couple of resonator tubes. A 250–fold enhancement of optical power was achieved inside the cavity, resulting in a LOD for CO of 2 ppb (3 σ, 10 s integration) [16].

Here, we report on an optimized I-QEPAS setup employing a T-shaped QTF with resonator tubes. Also, the stability and noise of the intracavity power buildup were improved using a feedback loop for optical feedback phase correction with increased bandwidth. Detection of CO, N_2_O and H_2_O is reported and compared with QEPAS measurements recorded without cavity enhancement with a spectrophone using the same T-shaped QTF. While the sensitivity of N_2_O and H_2_O increases linearly with the power enhancement, strong optical saturation of CO is observed. The performance of the setup is characterized and LODs in the ppt range have been achieved for both CO and N_2_O.

## 2 Experimental

The setup and principles of intracavity QEPAS with optical feedback were described previously [16], and additional details can be found in [17]. Here, the key components of the setup are briefly reviewed, and some details of the newly employed T-shaped tuning fork are provided.

### 2.1 Intracavity QEPAS with optical feedback

To achieve cavity enhancement of QEPAS signals, the quartz tuning fork (QTF) and resonator tubes are placed in the center of a Brewster window cavity. The cavity mirrors have a radius of curvature of 150 mm and a reflectivity of 0.9992 at 4.59 µm. Due to the smaller prong spacing of 0.8 mm of the T-shaped tuning fork, the 1/e^2^ beam waist radius is decreased as compared to [16] to 0.20 mm by increasing the cavity length to 29 cm. Light from a distributed feedback quantum cascade laser (DFB-QCL) is coupled into the cavity via reflection at a CaF_2_ window (“Brewster window”) placed in the cavity at an angle close to Brewster’s angle adjusted for maximum power buildup [16]. A half-wave plate and polarizer set the input polarization to parallel and a set of three mode-matching lenses overlaps the incoming beam with the fundamental mode of the cavity. Upon resonant power buildup, light is reflected back from the Brewster window and injected back into the DFB-QCL. This optical feedback locks the wavelength of the DFB-QCL to a cavity resonance, resulting in highly efficient and low noise buildup of optical power [18]. The optical power circulating in the cavity is modulated at the resonance frequency of the QTF by applying a ramped wavelength modulation to the DFB-QCL. The amplitude of wavelength modulation is chosen larger than the wavelength locking range for which optical feedback is effective, resulting in a switching between cavity-locked and free-running laser wavelength during every ramping period and an according modulation of intra-cavity power [10]. To achieve a stable power buildup with a fixed phase relative to the wavelength modulation waveform, the free-running laser center wavelength is fixed to a cavity resonance via an electronic feedback loop [16]. The error signal of the loop is generated from the demodulated signal from a photo detector recording a small fraction (~ 5%) of the light back reflected from the Brewster window, and feedback is provided through the laser current. To record QEPAS spectra, the laser center wavelength can be stepped and locked to consecutive cavity resonances while recording the QEPAS signal.

**Fig. 1:**
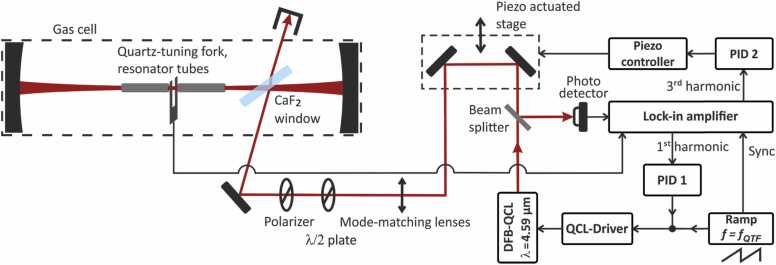
I-QEPAS setup, see text and [17]. λ/2 plate: half-wave plate; PID: feedback controller; f_QTF_: resonance frequency of quartz tuning fork; DFB-QCL: distributed feedback quantum cascade laser.

A second electronic feedback loop is necessary to control the optical phase of the re-injected light (“feedback-phase”). An error signal for this loop can be retrieved from the characteristic shape of the buildup signals recorded by the photo detector during ramped current modulation power [10]. Here, differing from [16], this is achieved by using the detector signal demodulated at the third harmonic of the ramping frequency. The loop is closed by adjusting the distance between the laser and the cavity. The demodulated signal is fed into a fast digital proportional-integral (PI) controller (Digilock 110, *Toptica*) whose output is connected to a piezo controller that moved two mirrors on a piezo-stage. As compared to the computer controlled feedback loop used in [16], the bandwidth of the new locking loop for the feedback phase is significantly higher (~ 50 Hz), yielding a strongly decreased noise floor of the modulated power inside the cavity that generated the photoacoustic signals. The signal on the photo detector (see Fig. 1) is directly proportional to the intracavity power and can hence be used to analyze the noise floor of the latter. With the new locking loop, the signal-to-noise ratio of the intra-cavity power, demodulated at the fundamental modulation frequency, was 3.3∙10^3^ (1 σ, 1 s integration time). The reduced buildup noise is critical to achieve the limits of detection reported below.

### 2.2 QEPAS module

In the first demonstration of the Fabry-Perot Brewster window cavity I-QEPAS setup, the selected QTF had the largest prong spacing (1.5 mm) among all the custom QTFs realized so far, with the aim of avoiding additional round-trip losses in the cavity and possible background noise due to light beam tails hitting the resonator [16]. Here, the employed spectrophone consists of a QTF with T-shaped prongs coupled with a pair of acoustic resonator tubes of optimized dimensions. Compared to QTFs with standard prongs having a rectangular longitudinal-section, QTFs with T-shaped prongs have been demonstrated to exhibit an increased stress field intensity, thus an enhanced generation of piezoelectric charges [6]. Moreover, when coupled with a pair of resonator tubes of 12.4 mm length and 1.59 mm inner diameter, the resulting spectrophone provides a record signal-to-noise ratio enhancement factor of 60, compared to the bare QTF [6]. As the most performant resonator[19], the T-shaped QTF was thus employed as photoacoustic transducer to further boost the I-QEPAS sensor performance in terms of detection sensitivity. However, since the prongs spacing is 0.8 mm, almost halved compared to [16], an undesired signal due to light power absorption at the prongs, i.e*.* background noise, could arise. The QEPAS module employed in this work includes the described spectrophone and a high-gain transimpedance amplifier transducing the QTF current into a voltage signal.

### 2.3 Sample preparation

Absorption lines of water vapor (2178.86 cm^−1^), carbon monoxide (2179.77 cm^−1^) and nitrous oxide (2179.28 cm^−1^) can be targeted within the emission spectral range of the employed laser source, by properly setting the laser operating temperature. Therefore, the I-QEPAS sensing performance was tested for the detection of H_2_O, CO and N_2_O. All measurements were performed in a steady gas flow of ~ 300 sccm. Water vapor acts as vibrational energy relaxation promoter on the excited levels of both carbon monoxide and nitrous oxide, resulting in a more efficient photoacoustic signal generation [20], [21]. Humidified CO and N_2_O mixtures were thus analyzed. A custom built gas mixing unit [22] was used to prepare samples of defined concentrations of CO and N_2_O from N_2_ 5.0 as well as premixed test gas cylinders of 1 ppm ± 5% of CO in N_2_ and 1 ppm ± 5% of N_2_O in N_2_ and N_2_. All concentrations of mixtures reported hereafter are nominal concentrations based on the specified concentrations of the test gas cylinders and the set mixing ratios. To the limits of the sensitivity of the I-QEPAS setup reported herein, no contamination of the pure N_2_ with CO, N_2_O or H_2_O could be observed. To set the humidity of the gas mixtures to a fixed value, a stream of diluted test gas was mixed with a stream of N_2_ that was saturated with water vapor in a wash bottle [16], [23]. A 2%_V_ H_2_O content was selected for both analytes mixtures, while optimized working pressures of 765 mbar and 490 mbar were employed for CO and N_2_O mixtures, respectively.

## 3 Results and discussion

### 3.1 Spectrum of H_2_O

**Fig. 2:**
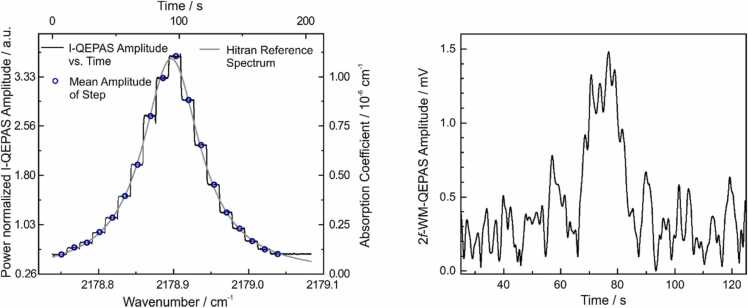
QEPAS spectra of H_2_O. Left: I-QEPAS spectrum of 0.7%_V_ H_2_O in N_2_, *p* = 480 mbar, see text. Right: 2*f*-WM-QEPAS spectrum of 1.9%_V_ H_2_O in N_2_, *p* = 500 mbar recorded with the same tuning fork without cavity enhancement.

Measurements on water vapor detection were performed to test the I-QEPAS setup and compare I-QEPAS sensing improvements with respect to a standard QEPAS setup employing the same laser source and spectrophone and operating under the same conditions. Fig. 2 shows an I-QEPAS (left panel) and a QEPAS (right panel) spectrum of a weak absorption line of H_2_O at 2178.9 cm^−1^, having an absorption coefficient α = 3 × 10^−6^ cm^−1^. The I-QEPAS spectrum for a mixture containing 0.7%_V_ H_2_O at 480 mbar was recorded by stepping the laser center wavelength to consecutive cavity resonances every ten seconds via the laser current. During every step, the laser center wavelength and optical feedback phase were locked via feedback loops and the I-QEPAS signal as well as the demodulated signal from the photo detector were recorded. The black solid line in the left hand panel in Fig. 2 shows the raw I-QEPAS signal versus time during a stepped wavelength scan. The I-QEPAS signal was normalized by the amplitude of the demodulated detector signal to correct for changes of the laser power with stepped laser current. The normalized I-QEPAS amplitude was averaged for every step (8 s, excluding the first and last second) and plotted on a wavenumber axis using the known free spectral range of 1/58 cm^−1^ of the cavity as the step width between consecutive data points. After fitting of the center wavelength, the recorded QEPAS spectrum resembles the reference spectrum of H_2_O simulated based on the HITRAN 2012 database [24]. Note that an offset signal of 0.26 a.u. was observed that could be attributed to absorption of intracavity power at the prongs of the tuning fork.

For comparison, the same QEPAS module and QCL were used in a 2 f-wavelength modulation (2 f-WM) experiment without cavity enhancement (compare right hand panel in Fig. 2). After optimizing the amplitude of wavelength modulation for maximum signal, a QEPAS spectrum was recorded for the same absorption line of water, but a higher concentration of 1.9%_V_. The 2 f-WM QEPAS signal in Fig. 2 is plotted on a time axis corresponding to a linear ramp of laser current. Wavelength calibration, e.g. based on known absorption line positions, is beyond the scope of this work.

The figures of merit for the two measurements are summarized in Table 1. The sensitivity of the raw I-QEPAS signal (without normalization) was calculated from the peak signal of 180 mV, from which an offset of 13 mV from absorption at the prongs of the QTF was subtracted. The sensitivity is 350 times higher than in the QEPAS experiment. This gain is directly related to the intra-cavity power buildup of ~ 100 (compare Section 3.2) and a scaling factor between intensity modulated (IM) and wavelength modulated (WM) photoacoustic excitation. Upon wavelength modulation, the absorbed optical power is distributed over different harmonics of the excitation frequency, including DC, and only a fraction of 34% (at optimum modulation parameters) is centered at the second harmonic [25]. For IM, this fraction can be significantly higher, depending on the excitation wave form (see also [26] for a comparison of IM and WM in QEPAS).

**Table 1:** Comparison of QEPAS and I-QEPAS measurements of water vapor.

	Peak signal [mV]	H_2_O concen-tration [%_V_]	Sensitivity [mV/%_V_]	Noise floor (1 σ) [mVHz^−1/2^]	SNR for 1%_V_ [Hz^1/2^]
QEPAS	1.3	1.9	0.68	0.27*	2.5^a^
I-QEPAS	180	0.7	240	0.15	1600

a influenced by excess noise, see text.

The noise floor of the 2 f-WM-QEPAS signal was measured as the standard deviation in the far wings of the absorption line and normalized by the square root of the detection bandwidth (0.2 Hz). The I-QEPAS noise floor is analyzed in Section 3.3. The higher noise of the 2 f-WM-QEPAS measurement originates from a too high gas flux through the spectrophone during the experiment. In principle, the same noise floor is expected in both experiments.

### 3.2 I-QEPAS of CO and N_2_O

Since the line strengths of the selected CO and N_2_O transitions are much higher than that of water and their relaxation dynamics are slower, it is important to verify if optical saturation arises due to the high laser intensity in the center of the cavity at the position of the QTF. To account for saturation, the I-QEPAS signal *S* can be described as [16], [17].

(1) $$S\left(I_{0}\right)=k\alpha_{0}\frac{\pi }{2}\sigma_{0}^{2}I_{\mathrm{sat}}\ln\left(1+\frac{I_{0}}{I_{\mathrm{sat}}}V\left(\nu -\nu_{0}\right)\right)$$

Herein, *I*_*0*_ is the peak intensity of the Gaussian intensity profile, *k* is a proportionality constant, *α*_0_ is the linear absorption coefficient at resonance, *σ*_0_ is the 1/e^2^ beam radius, *I*_*sat*_ is the saturation intensity and *V* is the peak-normalized line-shape function.

**Fig. 3:**
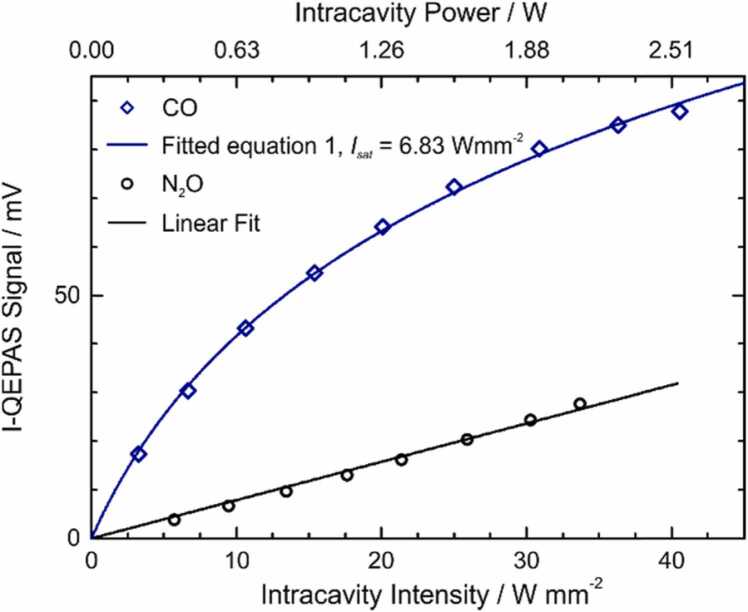
Scaling of I-QEPAS amplitude with optical power and intensity for CO and N_2_O. CO: 200 ppb in N_2_ and 2%_V_ H_2_O, *p* = 765 mbar. N_2_O: 200 ppb in N_2_ and 2%_v_ H_2_O, *p* = 490 mbar.

The scaling of *S* with *I*_*0*_ was investigated by targeting the CO absorption line at 2179.77 cm^−1^ (line strength of 4∙10^−19^ cm/molecule) and the N_2_O lines overlapping at 2179.28 cm^−1^ (strongest line strength of 6.7∙10^−20^ cm/molecule). The I-QEPAS signal was recorded for 200 ppb CO in N_2_ and 200 ppb of N_2_O in N_2_ while varying the laser power. In both cases, the H_2_O concentration was set to 2%_V_. The intracavity power was measured based on the ring-down time and cavity transmission as described in [16]. A power enhancement of the cavity of ~100 was measured, reduced as compared to previous experiments [16] due to additional round-trip losses at the more closely spaced prongs of the tuning fork (0.8 mm with respect to 1.5 mm in [16]). The recorded I-QEPAS signal was fitted with Eq. (1) using *k* and *I*_*sat*_ as fitting parameters and the result is shown in Fig. 3.

CO clearly shows an optical saturation effect, while a linear increase of the I-QEPAS signal with power confirms that N_2_O is not saturated at the present intensity.

### 3.3 Calibrations of CO and N_2_O signals

**Fig. 4:**
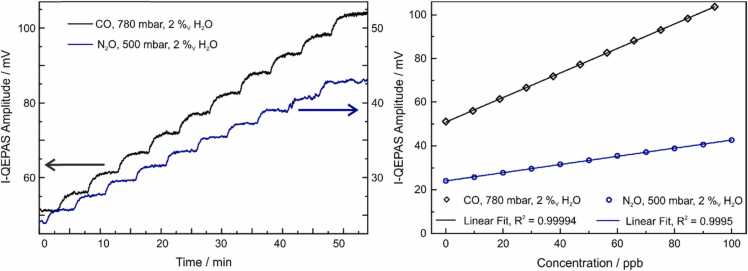
Calibration of I-QEPAS for CO and N_2_O. Left: Raw data recorded while increasing the concentration. Right: Calibration of mean I-QEPAS amplitude versus concentration.

To investigate the performance of the I-QEPAS setup for the detection of CO and N_2_O, calibration measurements were performed. For both CO and N_2_O, the concentration was increased stepwise in increments of 10 ppb from 0 ppb to 100 ppb while the laser was locked to a cavity resonance coinciding with the resonance frequency of CO (2179.77 cm^−1^) and N_2_O (2179.28 cm^−1^), respectively. The laser power imping on the cavity was 25 mW and 19.5 mW, respectively, for CO and N_2_O measurements, corresponding to the maximum intracavity power in Fig. 3. The raw signals and resulting linear calibrations are shown in Fig. 4.

The I-QEPAS signal of CO and N_2_O scale linearly with the gas concentration within the analyzed range, with a slope of 0.56 mV ppb^−1^ and 0.19 mV ppb^−1^, respectively. Note that the calibration is non-linear at higher concentrations when the molecular absorption significantly contributes to the cavity round-trip losses [16]. Offsets of 51 mV and 24 mV observed for CO and N_2_O, respectively, originate from absorption of the wings of the nearby water absorption line located at 2178.9 cm^−1^ as well as from absorption of optical power by the prongs of the QTF.

**Fig. 5:**
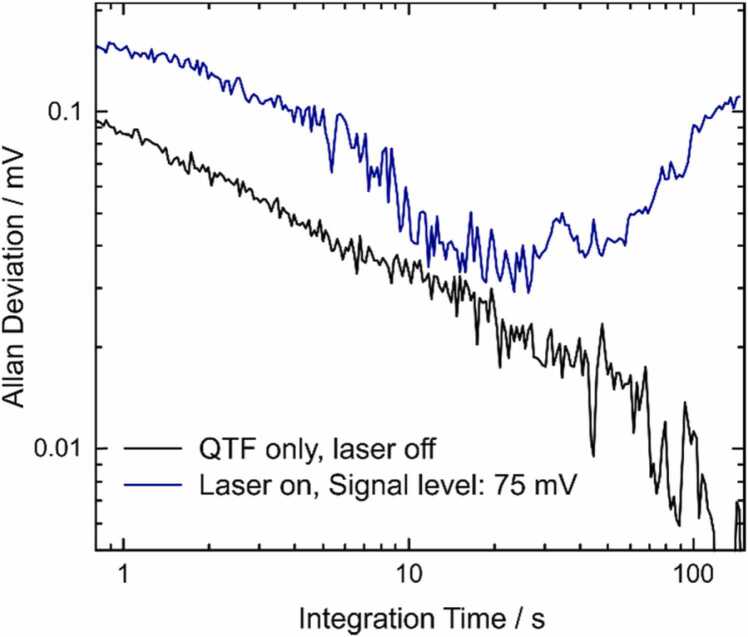
Allan-Werle Plot for I-QEPAS signals. Allan Deviation of the I-QEPAS amplitude recorded with the beam blocked (black line) and with the laser locked to a cavity resonance while the cell is filled with 94 ppb of CO in N_2_ and 2%_V_ at 490 mbar (blue line).

The noise floor was analyzed as a function of averaging time by means of Allan-deviation analysis [27]. The I-QEPAS signal of 94 ppb of CO in N_2_ and 2%_V_ H_2_O at 490 mbar (75 mV average I-QEPAS amplitude), as well as the signal from the QTF without laser excitation (0 mV average I-QEPAS amplitude) were recorded. Fig. 5 shows the Allan-Werle plots obtained from these measurements. The noise floor of the I-QEPAS signal of CO is ~ 1.5 times higher than that measured without laser excitation. The additional noise proportional to the I-QEPAS amplitude originates from buildup - noise of intracavity laser power. In the studied concentration range, this contribution is small due to highly reproducible and stable optical feedback locking and an actively stabilized optical feedback phase. For larger signals and longer integration time where the intracavity power may drift, noise from intracavity laser power can be reduced by normalizing signals to the laser power monitored on a photo detector (compare Section 3.1).

From the Allan deviation recorded with the laser turned on, the noise floor at 10 s integration time is 4.8∙10^−2^ mV, corresponding to a bandwidth normalized noise floor of 0.15 mV Hz^−1/2^. Considering the slopes of the calibration curves measured for CO and N_2_O, the related noise equivalent concentrations (NEC), limits of detection (LOD), noise equivalent absorption coefficient (NEA) and power normalized NEA (NNEA) were calculated. The results are summarized in Table 2. The use of an Intracavity-QEPAS setup employing a highly performant resonator (T-shaped QTF), combined with the stability of the intracavity power resulting from optical feedback locking with an optimized locking loop for the optical feedback phase, allowed us to achieve detection sensitivities in the ppt-range for both CO and N_2_O detection.

**Table 2:** Figures of merit of the I-QEPAS setup for detection of CO, N_2_O and H_2_O, see text.

	NEC / ppb Hz^−1/2^	LOD (3 σ, 10 s)	NEA / cm^−1^ Hz^−1/2^	NNEA / cm^−1^ W Hz^−1/2^
CO	0.27	260 ppt	1.5∙10^−8^ *	3.8∙10^−10^^a^
N_2_O	0.79	750 ppt	8.5∙10^−9^	1.7∙10^−10^
H_2_O	6300	5.9 ppm	1∙10^−9^	1.9∙10^−11^

a NEA and NNEA values for CO are affected by optical saturation.

NEA values were extracted from the NEC values based on reference spectra calculated using the HITRAN database [28]. The optical power entering the cavity used for calculating the NNEA was 25 mW, 19.5 mW and 19 mW for CO, N_2_O and H_2_O respectively (different for the three species due to different laser operating temperatures). Note that, since NNEA values imply a linear scaling with optical power, the value given for CO which is increased due to optical saturation is not directly comparable to values obtained in a linear absorption regime. The difference in NNEA of almost one order of magnitude between H_2_O and CO, N_2_O can be attributed to optical saturation of CO and the slower V-T transfer of CO [20] and N_2_O.

## 4 Conclusions

CO and N_2_O were measured at single-digit ppb concentrations using a cavity-enhanced QEPAS setup exploiting an optimized quartz tuning fork with T-shaped prongs and stable and low-noise optical feedback locking of a DFB-QCL to a high-finesse linear Brewster-window cavity. Optical feedback facilitates efficient coupling of optical power into the cavity, yielding an increase in effective optical power of ~ 100, without having a negative effect on the noise floor of QEPAS measurements. Limits of detection of 260 ppt and 750 ppt were achieved for CO and N_2_O, respectively, with an optical power available from the laser of 25 mW. The possibility to employ high power QCLs [7] emitting up to 1 W of optical power in combination with the realized intracavity QEPAS sensor would allow limits of detection in the low ppt range for many molecules, a sensitivity range currently reserved for TDLAS with large volume multipass cells or other cavity-enhanced and cavity ringdown techniques [29].

## Funding

This work has received funding from the COMET Center CHASE (project No. 868615), which is funded within the framework of COMET (Competence Centers for Excellent Technologies) by BMVIT, BMDW, and the Federal Provinces of Upper Austria and Vienna. The COMET program is run by the Austrian Research Promotion Agency (FFG). The authors from Dipartimento Interateneo di Fisica di Bari (Italy) and BL from TU Wien (Austria) acknowledge financial support from the European Union's Horizon 2020 research and innovation program via the Marie Skłodowska- Curie project OPTAPHI, grant No. 860808. MG, AS and VS also acknowledge financial support from THORLABS GmbH within the PolySenSe joint-research laboratory.

## Declaration of Competing Interest

No conflict of interest.
